# Comparison of the Monocyte-to-High-Density Lipoprotein Ratio and the Hemoglobin-to-Red Cell Distribution Width Ratio in Post-percutaneous Coronary Intervention (PCI) Coronary Heart Disease

**DOI:** 10.7759/cureus.108476

**Published:** 2026-05-08

**Authors:** Iqra Sheikh, Sharique Ahmad, Bashir Ahmad Mir, Andleeb Zehra, Sumaiya Irfan, Aparna Misra

**Affiliations:** 1 Department of Pathology, Era’s Lucknow Medical College and Hospital, Era University, Lucknow, IND; 2 Department of Cardiology, Era’s Lucknow Medical College and Hospital, Era University, Lucknow, IND; 3 Department of Biochemistry, Era’s Lucknow Medical College and Hospital, Era University, Lucknow, IND

**Keywords:** coronary artery disease, hemoglobin-to-red cell distribution width ratio, monocyte-to-high-density lipoprotein ratio, percutaneous coronary intervention, prognostic markers

## Abstract

Introduction: Coronary artery disease (CAD) is a major cause of morbidity and mortality worldwide, particularly in developing countries such as India. Although percutaneous coronary intervention (PCI) has improved the management of coronary heart disease (CHD), post-PCI outcomes continue to be influenced by inflammatory and hematological parameters. Novel indices, including the hemoglobin-to-red cell distribution width ratio (HRR) and the monocyte-to-high-density lipoprotein ratio (MHR), have emerged as potential markers of inflammation, oxidative stress, and cardiovascular outcomes. This study aimed to evaluate the short-term prognostic association of HRR and MHR in patients with CHD undergoing PCI. The primary objective was to assess their relationship with six-month post-PCI clinical outcomes, while secondary objectives included evaluation of mortality, mild symptoms, composite poor outcome, and exploratory receiver operating characteristic (ROC)-derived cutoff values.

Methodology: This prospective cohort study included 138 patients with CHD who underwent PCI at Era’s Lucknow Medical College and Hospital. Hematological and lipid parameters were analyzed using automated techniques, and HRR and MHR were calculated. Patients were followed for six months to assess post-PCI outcomes, including mild symptoms and mortality. Statistical analyses were performed using the independent-samples t-test, analysis of variance, chi-square test, and ROC curve analysis. Because of the limited cohort size and low number of mortality events, ROC-based mortality analysis was considered exploratory, and no multivariable regression model was performed.

Results: The mean age of the patients was 56.64 ± 10.05 years. Of the 138 patients, 103 (74.6%) were male and 35 (25.4%) were female patients. HRR abnormality (≤1.0) was observed in 82/138 patients (59.4%), while MHR abnormality (≥0.14) was observed in 21/138 patients (15.2%). After PCI, 117/138 patients (84.8%) remained asymptomatic, 17/138 (12.3%) developed mild symptoms, and 4/138 (2.9%) died during follow-up. HRR values were significantly higher in male than female patients, whereas MHR did not differ significantly by sex. Elevated MHR was significantly associated with adverse post-PCI outcomes (p < 0.001). ROC analysis showed exploratory discriminative ability for mortality, with AUCs of 0.838 for HRR and 0.863 for MHR. These AUC values were derived internally from a small number of mortality events and should therefore be interpreted with caution.

Conclusion: Both HRR and MHR demonstrated short-term unadjusted prognostic associations in post-PCI CHD patients, with MHR showing numerically higher exploratory discriminatory performance for adverse outcomes. Larger studies with longer follow-up are needed to validate these findings. External validation and adequately powered multivariable analyses are required before HRR and MHR can be considered independent predictors or clinically generalizable thresholds. Given the small cohort, low mortality-event count, short follow-up, and absence of multivariable adjustment, these findings should be interpreted as preliminary and hypothesis-generating rather than definitive evidence for clinical risk stratification.

## Introduction

Coronary artery disease (CAD) is a common cardiac condition characterized by atherosclerotic plaque in the coronary artery lumen [1]. Although often asymptomatic, it remains a leading cause of mortality and disability worldwide [2]. In the United States, CAD accounts for over 600,000 deaths annually, nearly one-fourth of total deaths [3]. The 2016 Heart Disease and Stroke Statistics update of the American Heart Association reported that 15.5 million persons aged ≥20 years in the USA have coronary heart disease (CHD) [4].

Globally, cardiovascular disease caused nearly 20 million deaths in 2022 [3]. Low- and middle-income countries (LMICs) like India bear a heavy burden, with nearly seven million deaths annually due to CAD and 129 million disability-adjusted life years (DALYs) lost in LMICs alone [3]. In 2015, CAD accounted for 8.9 million deaths and 164 million DALYs worldwide [5]. India not only has one of the highest incidences of cardiovascular disease, including CAD, but also has one of the fastest growth rates. CHD accounted for 17% of total and 26% of adult deaths in 2001-2003, increasing to 23% and 32% during 2010-2013 [6]. Prevalence of CAD has risen from 1% to 9%-10% in urban areas and from <1% to 4%-6% in rural India [6].

Rapid lifestyle changes have made India a major contributor to the global heart disease burden, nearly 60% of the world’s cases. The average age of onset in Indian patients is five to eight years earlier than in developed countries, with ischemic heart disease predominating [7]. Percutaneous coronary intervention (PCI) is a preferred nonsurgical procedure to restore blood flow in occluded coronary arteries [8]. It involves balloon angioplasty followed by stent deployment to maintain arterial patency [9]. Despite its effectiveness, restenosis may occur due to persistent inflammatory and metabolic factors [10]. Atherosclerosis, the underlying pathology, involves plaque accumulation, inflammation, and thrombosis, which can trigger acute coronary events [11].

While several biochemical and imaging biomarkers have been proposed for predicting CAD outcomes, most are costly and not readily available [12]. Simple hematological parameters, such as red cell distribution width (RDW), monocyte count, and hemoglobin, have gained attention for prognostic value [13]. Elevated RDW is associated with poor outcomes in heart failure, myocardial infarction, and post-PCI cases [14,15]. Novel indices, such as the monocyte-to-high-density lipoprotein ratio (MHR) and the hemoglobin-to-RDW ratio (HRR), have emerged as reliable markers of inflammation and oxidative stress [15,16].

MHR reflects the balance between monocyte-mediated inflammatory activity and the anti-inflammatory, antiatherogenic effect of high-density lipoprotein, whereas HRR reflects the combined influence of hemoglobin status and RDW, both of which may be affected by anemia, systemic inflammation, oxidative stress, and impaired erythropoiesis. These mechanisms are clinically relevant after PCI because inflammation, oxidative stress, anemia, and lipid-related vascular dysfunction may influence restenosis, recurrent ischemic events, postprocedural recovery, and mortality. Therefore, the present study was planned to compare the novel predictive markers MHR and hemoglobin-to-red-cell distribution width ratio (HRR) in CHD patients post-PCI. Given the limited cohort size and six-month follow-up period, the study was designed to assess short-term prognostic associations rather than to establish definitive independent prediction or long-term clinical thresholds.

Objectives of the study

The primary objective of this prospective cohort study was to assess the association between baseline HRR and MHR and short-term post-PCI clinical outcomes during a six-month follow-up period among patients with CHD.

The secondary objectives were 1) to compare HRR and MHR across demographic subgroups, particularly age and sex; 2) to evaluate the relationship of HRR and MHR with individual post-PCI outcomes, including asymptomatic status, mild symptoms, and mortality; 3) to assess their association with a composite poor outcome defined as mild symptoms or mortality during follow-up; and 4) to derive exploratory study-specific receiver operating characteristic (ROC)-based cutoff values for mortality and composite poor outcomes. These ROC-derived cutoffs were intended for exploratory analysis only and were not considered externally validated clinical thresholds. The study therefore evaluated unadjusted short-term prognostic associations and discriminatory performance, rather than confirming HRR and MHR as independent predictors of long-term mortality, restenosis, or major adverse cardiovascular events (MACE).

## Materials and methods

Study design and setting

The present study was conducted as a prospective cohort study at the Department of Pathology in collaboration with the Departments of Cardiology and Biochemistry, Era’s Lucknow Medical College and Hospital, Lucknow. The institution is a tertiary care center equipped with advanced facilities and primarily serves socioeconomically underprivileged suburban and rural populations of Lucknow. The study was designed as an exploratory single-center cohort study to assess the short-term prognostic association of HRR and MHR among post-PCI CHD patients.

Patients were followed for six months after PCI; therefore, the study primarily evaluated short-term post-PCI outcomes rather than long-term prognostic endpoints. This follow-up duration was insufficient to comprehensively assess clinically meaningful long-term outcomes, including late mortality, restenosis, stent thrombosis, recurrent myocardial infarction, repeat revascularization, and MACE. Therefore, the findings were interpreted with appropriate caution, particularly for mortality-based analyses, because of the limited number of observed mortality events during follow-up. Accordingly, the prognostic implications of HRR and MHR should be considered preliminary and require validation through larger studies with extended follow-up periods to determine their association with long-term mortality, restenosis, and MACE.

Duration and sample size

The study was conducted over 24 months, from April 2022 to April 2024. A total of 138 patients diagnosed with CHD were enrolled. Sample size was calculated using ROC curve analysis based on the expected AUC of 0.619 from a previous study [12], with 95% confidence interval and 80% power. The minimum required sample size was estimated to be 138. Although this sample size met the minimum estimated requirement for the planned analysis, the cohort remained relatively small, with only four mortality events recorded during the six-month follow-up period. This low event count reduces the statistical power for mortality-specific comparisons and may limit the stability and precision of ROC-derived estimates. Accordingly, mortality-related AUC values and study-specific cutoff points should be regarded as preliminary and hypothesis-generating rather than definitive prognostic thresholds.

Sampling frame and selection criteria

Patients diagnosed with CHD who attended the Department of Cardiology after confirmation by echocardiography and Troponin-I investigations were included. Eligible patients were screened from the Department of Cardiology during the study period after clinical diagnosis of CHD and planned PCI. Patients who fulfilled the inclusion criteria and provided written informed consent were enrolled. Inclusion criteria comprised patients with CHD confirmed by ECG, echocardiography, and Troponin-I testing who were scheduled to undergo PCI. The diagnosis and decision to perform PCI were made by the treating cardiology team based on clinical assessment and institutional practice.

Exclusion criteria included patients with malignant tumors, infectious or hematological diseases, severe heart failure, congenital, rheumatic, or valvular heart disease, as well as severe hepatic or renal dysfunction. These exclusions were applied to reduce the influence of conditions that could independently alter hematological indices, inflammatory status, lipid parameters, or post-PCI outcomes. Detailed clinical presentation categories, such as acute coronary syndrome vs. stable CAD, and detailed procedural variables, including lesion complexity, stent type, the number of vessels treated, and medication adherence, were not systematically incorporated into adjusted analyses.

Ethical considerations

The study protocol received approval from the Institutional Ethical Committee of Era’s Lucknow Medical College and Hospital, Lucknow. Written informed consent was obtained from all participants before inclusion in the study.

Methodology

Four milliliters of venous blood were collected from each patient, half in an ethylenediaminetetraacetic acid (EDTA) vial and half in a plain vial. Blood samples were collected at baseline during the index hospital evaluation before post-PCI follow-up outcome assessment. Samples in EDTA vacutainers were analyzed for hematological parameters using a Transasia H 560 Automated Blood Cell Count Analyzer (XS-800i; Transasia Bio-Medicals Ltd., Mumbai, India), with manual cross-checking by microscopy for accuracy. Plain-vial samples were used for biochemical analysis, including lipid parameters required to calculate MHR.

The parameters assessed included hemoglobin, red cell distribution width coefficient of variation (RDW-CV), and monocyte count from the differential leukocyte count. From these values, two ratios were calculated: hemoglobin-to-RDW-CV ratio (HRR) and MHR. HRR was calculated by dividing hemoglobin by RDW-CV, and MHR was calculated by dividing monocyte percentage by high-density lipoprotein level. Hemoglobin was measured using a colorimetric method. The baseline voltage of the diluent was recorded before sample addition. After mixing the blood with lysing reagent for complete reaction, the sample voltage was measured, and the hemoglobin concentration was determined based on Lambert-Beer’s law.

For interpretation, a lower HRR value reflects lower hemoglobin relative to RDW and may indicate anemia, ineffective erythropoiesis, or greater red cell size variability, whereas a higher MHR value reflects increased monocyte-mediated inflammation relative to HDL and may indicate a heightened inflammatory and oxidative stress state. In the present study, HRR ≤1.0 and MHR ≥0.14 were considered abnormal for categorical analysis. These predefined categorical thresholds were used for descriptive and group-wise comparison within the present cohort. Separately, ROC analysis was performed to generate study-specific exploratory cutoff values for mortality and composite poor outcomes using the Youden index. Therefore, the categorical thresholds and ROC-derived thresholds served different analytical purposes and should not be interpreted interchangeably.

In ROC analysis, study-specific cutoff values were derived to predict mortality and poor outcomes. Because mortality events were few, ROC analysis for mortality was performed as an exploratory assessment. The resulting AUC values, sensitivity, specificity, and cutoff values were interpreted as internally derived estimates requiring external validation in larger cohorts with adequate event numbers.

Follow-up and outcomes

All patients were followed up for six months post-PCI. Follow-up information was obtained from available clinical records and post-PCI follow-up documentation. The primary outcome for the present analysis was short-term post-PCI clinical status at six months, categorized as asymptomatic status, mild symptoms, or mortality. A composite poor outcome was defined as mild symptoms or mortality. Although MACE-related endpoints such as restenosis, repeat revascularization, recurrent myocardial infarction, and stent thrombosis are clinically important, these were not comprehensively captured in the reported results and were therefore not analyzed as formal primary endpoints.

Mild symptoms were defined as patient-reported fatigue, anxiety, or chest pain during follow-up that did not require hospital admission or urgent repeat intervention. Mortality was defined as death recorded during the six-month follow-up period. Demographic and clinical data were collected from patient case files and recorded on a structured sheet. Laboratory findings and follow-up outcomes were also documented until discharge or outcome. Formal blinded outcome adjudication was not performed, and outcomes were assessed using available clinical documentation. Given the short follow-up duration and the low number of deaths, the study was not powered to make robust mortality-specific conclusions. Therefore, mortality findings were considered supportive of a possible association rather than conclusive evidence of independent predictive performance.

Statistical analysis

The data were analyzed using Statistical Package for the Social Sciences version 25.0 software (IBM Corp., Armonk, NY). Qualitative/categorical data were presented as numbers and percentages, and continuous data were presented as mean ± standard deviation. The chi-square test, independent-samples t-test, and one-way analysis of variance (ANOVA), followed by Tukey’s honestly significant difference post hoc test, were used to compare data, as appropriate. Normality assumptions for continuous variables were assessed before applying parametric tests where applicable. ROC curve analysis was performed to derive study-specific cutoff values of predictors. The Youden index was used to identify exploratory ROC-derived cutoff values by maximizing the combined sensitivity and specificity within the present dataset. A p value of <0.05 was considered statistically significant. Considering the small cohort size and the very low mortality event count, no multivariable mortality model was attempted, as such modeling would be statistically unstable and prone to overfitting. Multivariable regression analysis was also not performed for overall adverse outcomes because the number of clinically relevant events was limited, and the available dataset did not include complete adjustment variables for all major potential confounders.

Important covariates such as age, diabetes mellitus, hypertension, renal function, baseline lipid status, smoking history, clinical presentation as acute coronary syndrome versus stable CAD, lesion complexity, stent type, number of diseased vessels, procedural details, and post-PCI pharmacotherapy may influence outcomes after PCI. The absence of adjustment for these confounders limits the ability to determine whether MHR and HRR are independent predictors of post-PCI outcomes. ROC-derived mortality estimates were therefore reported descriptively and interpreted with caution, acknowledging the possibility of overestimation of predictive performance. The observed associations should therefore be interpreted as unadjusted associations rather than evidence of independent prognostic value. Larger multicenter studies with longer follow-up and higher event numbers are required to evaluate and validate the prognostic relevance of HRR and MHR. Future studies should incorporate adequately powered multivariable Cox proportional hazards or logistic regression models to evaluate whether HRR and MHR remain independently associated with mortality, restenosis, and MACE after adjustment for established demographic, clinical, biochemical, and procedural risk factors.

## Results

Baseline demographic characteristics

**Figure 1:**
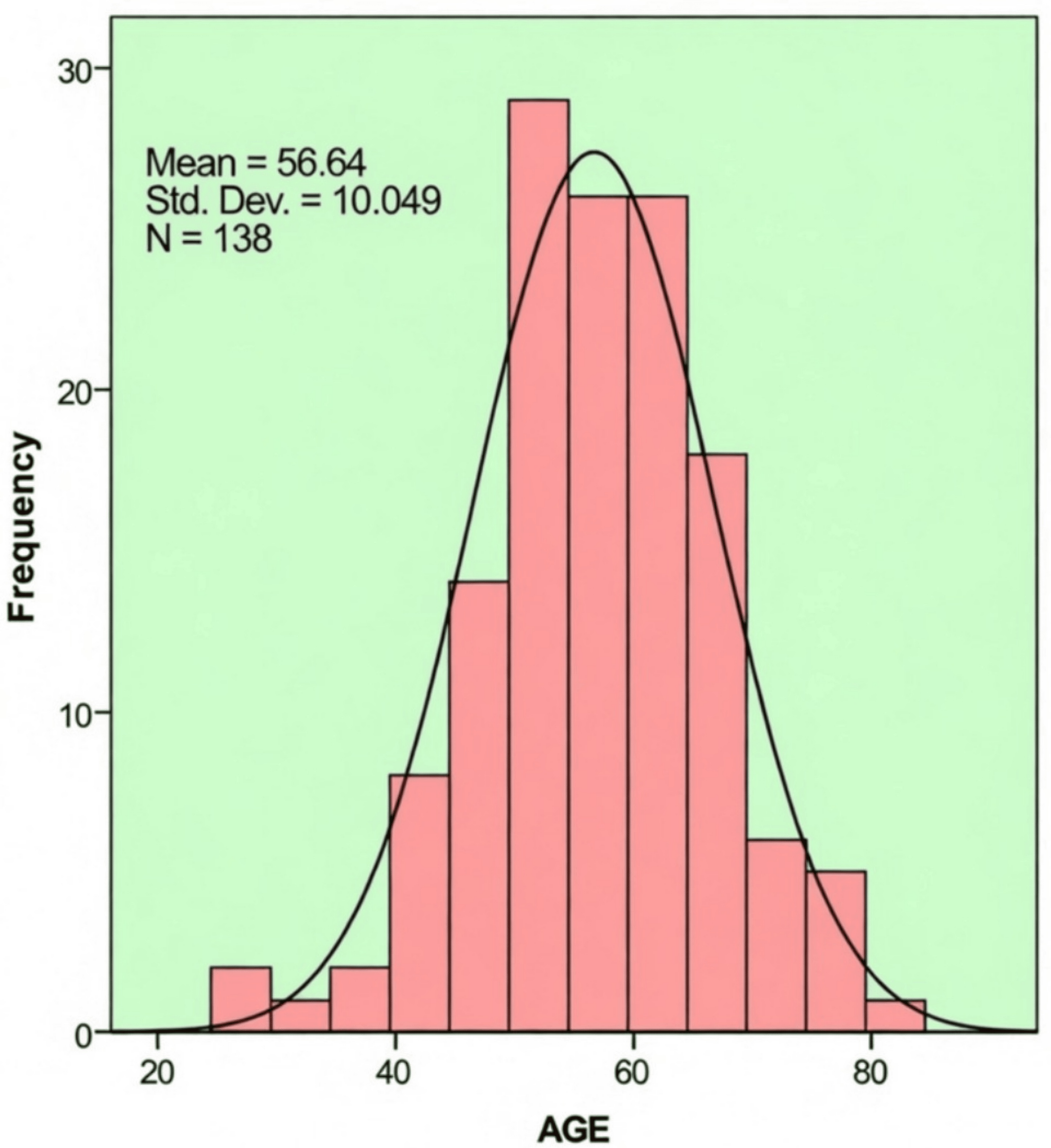
Age-wise distribution of study population (n = 138)

The age of patients ranged from 27 to 84 years, with a mean age of 56.64 ± 10.05 years. The most common age groups were 51-60 years (37.0%) and 61-70 years (28.3%), while ≤40 years (5.1%) and >70 years (6.5%) were the least common (Figure 1).

**Figure 2:**
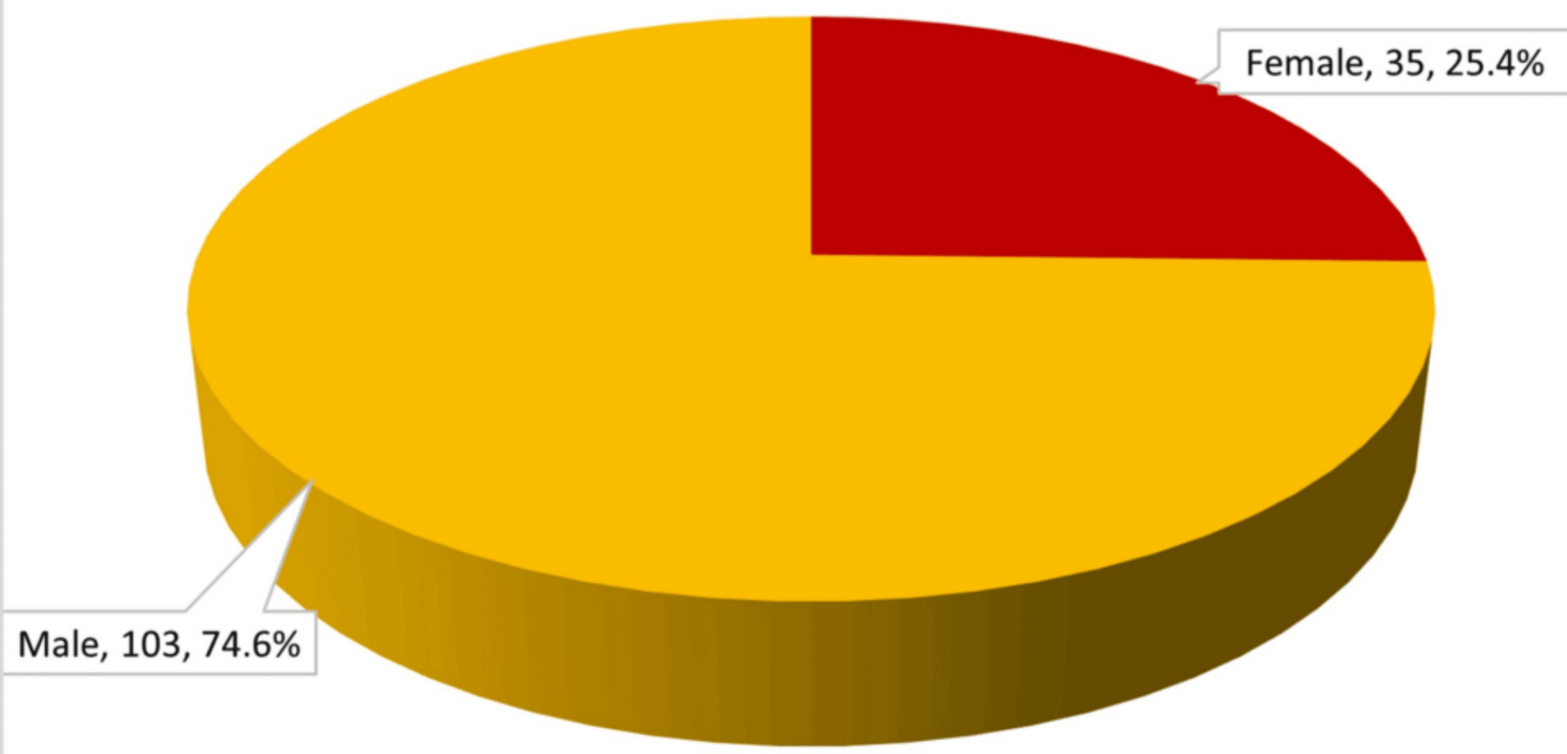
Gender-wise distribution of study population (n = 138)

Among 138 patients, 25.4% were female and 74.6% were male patients, with a male-to-female ratio of 2.94 (Figure 2).

Hematological and biochemical parameters

The mean and range values of key parameters, including hemoglobin, RDW, HDL, monocytes, MHR, and HRR, are shown in Table 1.

**Table 1:** Hematological parameters and novel markers in the study population (n = 138) SD: standard deviation

Variables	Min.	Max	Mean	SD
Serum hemoglobin (g/dL)	6.10	16.20	11.91	1.82
Red cell distribution width (%)	11.0	16.1	12.52	1.10
High-density lipoprotein levels (mg/dL)	14	78	36.52	8.79
Monocytes (%)	1	9	3.17	1.51
Monocyte-to-high-density lipoprotein ratio	0.0200	0.2727	0.0918	0.0496
Hemoglobin-to-red cell distribution ratio	0.5169	1.3304	0.9579	0.1600

**Figure 3:**
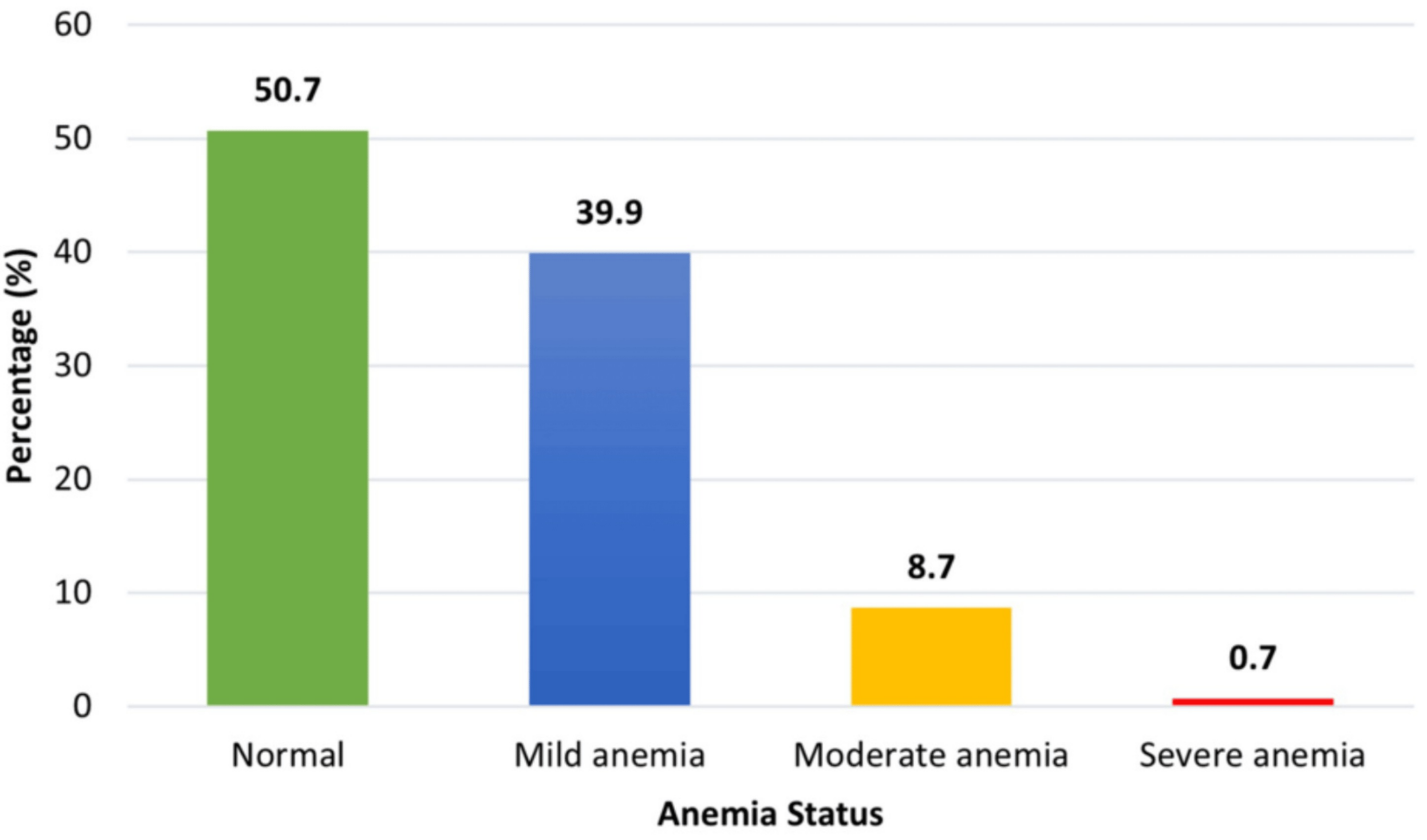
Anemic status of study population (n = 138)

Nearly half (50.7%) of the patients had no anemia, 39.9% had mild anemia, 8.7% had moderate anemia, and 0.7% had severe anemia (Figure 3).

**Figure 4:**
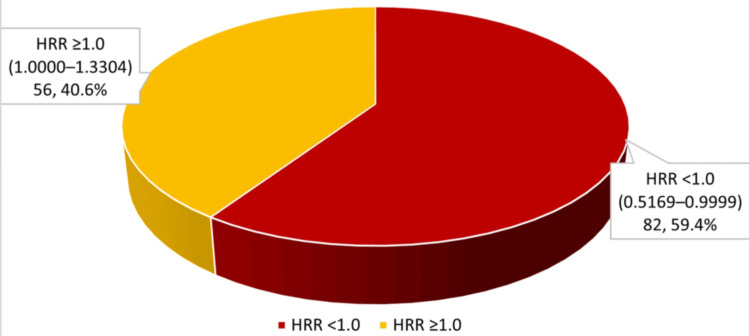
Distribution of HRR levels in study population (n = 138) HRR: hemoglobin-to-red cell distribution width ratio

**Figure 5:**
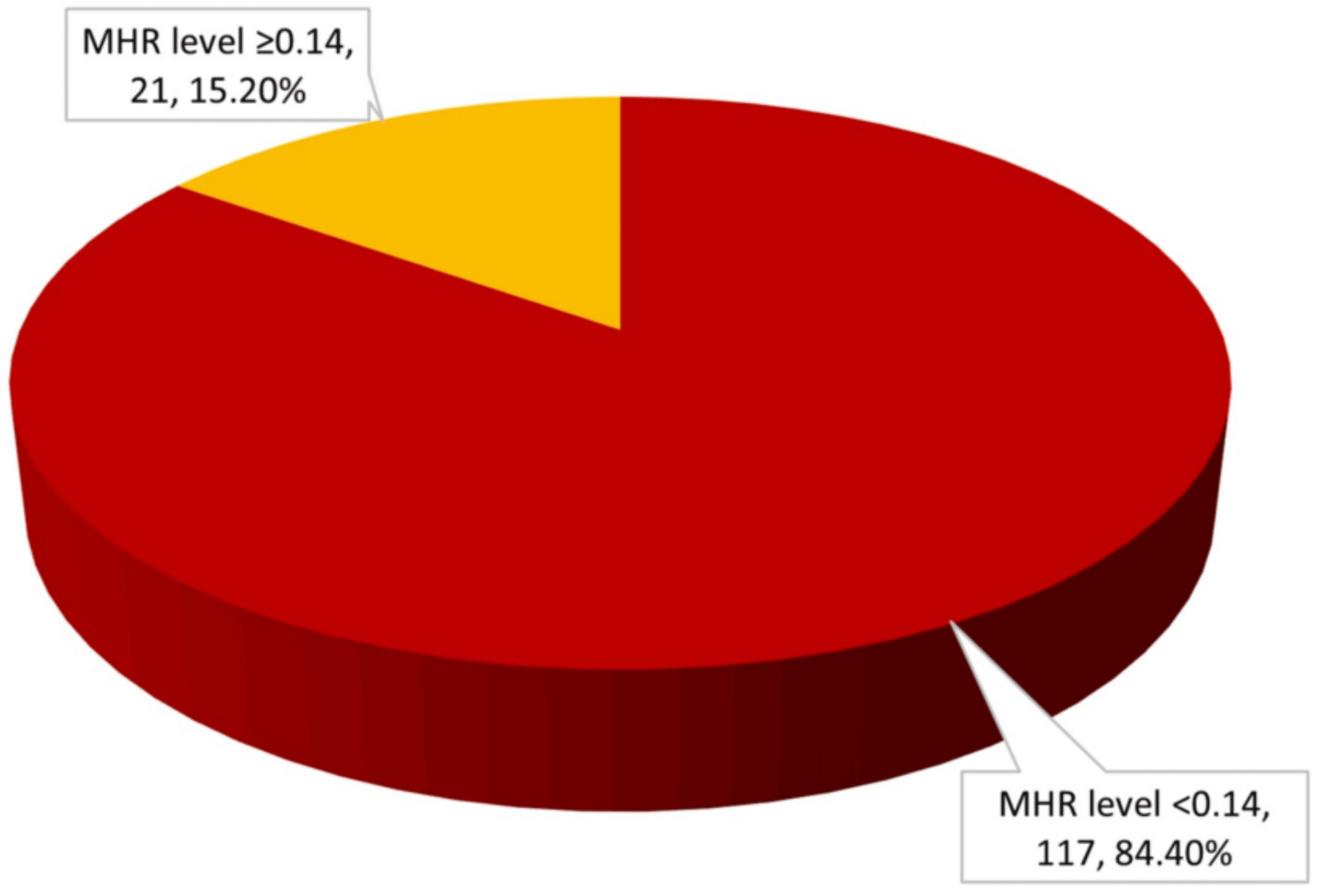
Distribution of MHR levels in study population (n = 138) MHR: monocyte-to-high-density lipoprotein ratio

HRR abnormality (<1.0) was noted in 59.4% of patients, while MHR abnormality (≥0.14) was observed in 15.2% (Figures 4, 5).

Post-PCI clinical outcomes

**Figure 6:**
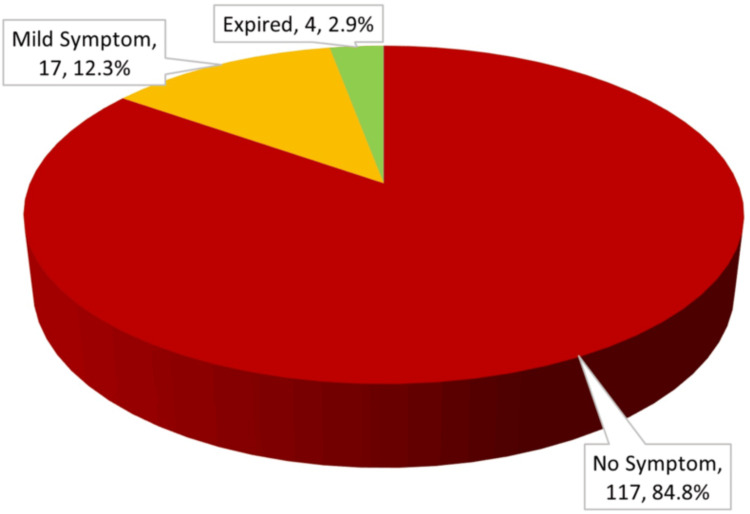
Post-PCI outcome distribution in study population (n = 138) PCI: percutaneous coronary intervention

Following PCI, 84.8% of patients remained asymptomatic, 12.3% developed mild symptoms, and 2.9% expired during follow-up (Figure 6).

Association of HRR and MHR with age

Following PCI, 84.8% had no adverse symptoms, 12.3% had mild symptoms, and 2.9% expired (Figure 6). HRR values were higher among younger patients than older ones, though not statistically significant (p = 0.062). Similarly, MHR was higher in younger patients than in older groups, with no significant difference (p = 0.261) (Table 2).

**Table 2:** Association of age with HRR and MHR (n = 138) ANOVA test has been used HRR: hemoglobin-to-red cell distribution ratio; MHR: monocyte-to-high-density lipoprotein ratio; ANOVA: analysis of variance; SD: standard deviation

Age group (years)	No. of patients (n = 138)	HRR			MHR		
		Mean ± SD	F	p	Mean ± SD	F	p
≤40	7	1.0405 ± 0.1703	2.305	0.062	0.1291 ± 0.0498	1.334	0.261
41-50	32	0.9893 ± 0.1183			0.0957 ± 0.0474		
51-60	51	0.9435 ± 0.1792			0.0857 ± 0.0454		
61-70	39	0.9643 ± 0.1469			0.0923 ± 0.0569		
>70	9	0.8351 ± 0.1749			0.817 ± 0.0419		

Association of HRR and MHR with gender

The mean HRR in male patients was significantly higher than in female patients. Mean MHR was also higher in male patients than female patients, but not significantly (p = 0.064) (Table 3).

**Table 3:** Association of gender with HRR and MHR (n = 138) Student's t-test was used ^*^p < 0.05 was considered statistically significant HRR: hemoglobin-to-red cell distribution ratio; MHR: monocyte-to-high-density lipoprotein ratio; SD: standard deviation

Gender	No. of patients (n = 138)	HRR			MHR		
		Mean ± SD	t	p	Mean ± SD	t	p
Female	35	0.8617 ± 0.1442	4.380	0.001^*^	0.0784 ± 0.0402	1.866	0.064
Male	103	0.9905 ± 0.1523			0.0964 ± 0.0519		

HRR and MHR in relation to clinical outcomes

HRR was maximum among asymptomatic patients (0.9666 ± 0.1582). MHR showed an opposite trend, being highest in expired patients (0.1686 ± 0.0662) (Table 4).

**Table 4:** Association of post-PCI outcome with HRR and MHR (n = 138) The ANOVA test was used ^*^p < 0.05 was considered statistically significant HRR: hemoglobin-to-red cell distribution ratio; MHR: monocyte-to-high-density lipoprotein ratio; PCI: percutaneous coronary intervention; ANOVA: analysis of variance; SD: standard deviation

Post-PCI outcome	No. of patients (n = 138)	HRR			MHR		
		Mean ± SD	F	p	Mean ± SD	F	p
No symptom	117	0.9666 ± 0.1582	3.960	0.021^*^	0.0861 ± 0.0427	7.883	0.001^*^
Mild symptom	17	0.9479 ± 0.1399			0.1134 ± 0.0699		
Expired	4	0.7437 ± 0.1793			0.1686 ± 0.0662		

Patients with HRR ≤1.0 had a higher incidence of mild symptoms and mortality than those with HRR >1.0, although the association was not statistically significant. Conversely, patients with MHR ≥0.14 had higher proportions of mild symptoms and deaths, indicating an unadjusted association between elevated MHR and adverse post-PCI outcomes (Table 5).

**Table 5:** Association of HRR (≤1.0) and MHR (≥0.14) abnormalities with post-PCI outcome (n = 138) Chi-square test was used ^*^p < 0.05 was considered statistically significant HRR: hemoglobin-to-red cell distribution ratio; MHR: monocyte-to-high-density lipoprotein ratio; PCI: percutaneous coronary intervention

Post-PCI outcome	HRR				MHR			
	>1.0 (n = 56)	≤1.0 (n = 82)	χ²	p	≤0.14 (n = 117)	>0.14 (n = 21)	χ²	p
No symptom	51 (43.6%)	66 (56.4%)	4.051	0.132	104 (88.9%)	13 (11.1%)	15.265	0.001^*^
Mild symptom	5 (29.4%)	12 (70.6%)			12 (70.6%)	5 (29.4%)		
Expired	0 (0.0%)	4 (100.0%)			1 (25.0%)	3 (75.0%)		

ROC analysis

ROC analysis showed exploratory discriminative ability of HRR for mortality, with an AUC of 0.838 at a cutoff ≤0.8116. MHR showed a numerically higher AUC of 0.863, at a cutoff ≥0.1017. For overall poor outcomes, HRR had an AUC of 0.605, while MHR had limited-to-moderate exploratory discriminatory ability (AUC = 0.655) (Table 6). Because only four mortality events occurred during follow-up, these ROC-derived estimates should be interpreted cautiously as internally derived, hypothesis-generating measures rather than stable or externally validated predictive-performance estimates.

**Table 6:** ROC analysis of HRR and MHR for exploratory discrimination of mortality and poor outcomes (n = 138) ^*^p values indicate whether the AUC is significantly different from 0.5, and the Youden index (J) was used to determine optimal cutoff values, which were not calculated for nonsignificant results AUC: area under the curve; CI: confidence interval; HRR: hemoglobin-to-red cell distribution ratio; MHR: monocyte-to-high-density lipoprotein ratio; ROC: receiver operating characteristic

Outcome type	Variable	AUC (95% CI)	p value	Youden index (J)	Projected cutoff value	Sensitivity (%)	Specificity (%)
Mortality	HRR	0.838 (0.667-1.000)	0.022^*^	0.611	≤0.8116	75.0	85.1
	MHR	0.863 (0.742-0.985)	0.014^*^	0.672	≥0.1017	100.0	67.2
Poor outcome (mild symptom + mortality)	HRR	0.605 (0.474-0.736)	0.126	-	Not applicable	-	-
	MHR	0.655 (0.510-0.800)	0.024^*^	0.339	≥0.1324	47.6	86.3

**Figure 7:**
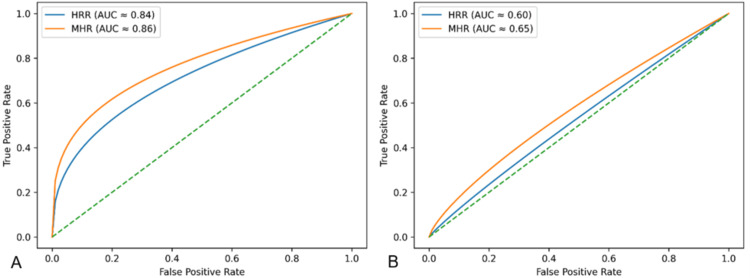
ROC curve for exploratory discrimination of (A) mortality and (B) poor outcome using HRR and MHR (n = 138) ROC: receiver operating characteristic; HRR: hemoglobin-to-red cell distribution width ratio; MHR: monocyte-to-high-density lipoprotein ratio; AUC: area under the curve

Figure 7 illustrates ROC curves for HRR and MHR, showing that MHR had numerically higher AUC values than HRR for mortality and overall poor outcomes in the present cohort. However, this finding should be interpreted with caution due to the low number of mortality events, the short follow-up duration, the internally derived cutoff values, and the lack of external validation.

## Discussion

CHD involves the narrowing of coronary arteries due to atherosclerosis, reducing blood flow and increasing risks of angina or myocardial infarction. In the present study, ROC-derived cutoff values were calculated to explore the discriminatory ability of HRR and MHR for mortality and poor post-PCI outcomes within this specific cohort. These thresholds were not selected as universal clinical cutoffs but were generated statistically using the Youden index to identify the point that maximized sensitivity and specificity in the study population. Therefore, the proposed cutoff values should be interpreted as internally derived, study-specific estimates rather than definitive prognostic thresholds. Their applicability to other populations may be limited because HRR and MHR can be influenced by demographic profile, anemia burden, inflammatory status, lipid levels, comorbidities, clinical presentation, and treatment-related factors.

The absence of external validation is an important limitation. Without validation in an independent cohort, the ROC-derived thresholds may overestimate predictive performance and may not retain the same sensitivity, specificity, or prognostic accuracy in broader clinical settings. Therefore, these cutoffs should not be used as definitive decision-making thresholds at this stage. Instead, they provide preliminary reference values that may guide future research. Larger multicenter studies with independent validation cohorts are required to determine whether these thresholds are reproducible and clinically generalizable.

Post-PCI outcomes are influenced by patient factors, disease severity, and procedural quality. Older patients with comorbidities often face higher risks of restenosis and stent thrombosis [17]. Patients presenting with acute coronary syndromes, particularly ST-elevation myocardial infarction, have greater risks of heart failure, recurrent infarction, and mortality [18]. Anatomical complexity, lesion characteristics, and completeness of revascularization also affect prognosis [19]. Procedural factors such as stent type and optimal deployment further influence outcomes. Drug-eluting stents reduce restenosis but require prolonged dual antiplatelet therapy [20,21]. Adherence to dual antiplatelet therapy post-PCI is crucial, as nonadherence increases thrombotic risk [22]. In the present study, detailed procedural characteristics, including lesion complexity, stent type, number of vessels treated, completeness of revascularisation, and post-PCI medication adherence, were not incorporated into adjusted analyses. This limits the ability to determine whether the observed associations of HRR and MHR with post-PCI outcomes were independent of procedural and treatment-related factors.

Genetic and inflammatory markers can further modulate outcomes [23,24]. Given the multifactorial nature of post-PCI outcomes, no single marker reliably predicts prognosis. Therefore, the present study focused on two novel hematological indices, MHR and HRR, to assess their predictive value in CHD patients post-PCI. For this purpose, a total of 138 patients meeting the eligibility criteria (age 27-84 years; mean 56.64 ± 10.05 years; 74.6% men) were enrolled. Compared to our study, Wu et al. carried out their study on 673 CAD patients (mean age = 59.1 years; 80.7% men) [25]. Xiu et al. included 6,046 patients (74.3% male patients; mean age ~60 years) [26], and Çiçek et al. studied 682 patients (mean age = 56.6 years; >80% male patients) [27].

Thus, although the age and sex profiles were similar, sample sizes in other studies were much larger, which represents a limitation of the present study. The present study followed patients for six months post-PCI, which constitutes short-term follow-up. Other studies had longer follow-ups, with Wu et al. reporting 31-45 months [25] and Xiu et al. reporting a mean follow-up of 35.9 months [26]. Therefore, follow-up duration is another limitation of the present study. In the present study, 40.6% of patients had HRR >1.0. In comparison, Xiu et al. reported higher HRR in 61% of patients, indicating differences in cohort characteristics [26]. We found higher HRR to be significantly associated with male sex, consistent with Xiu et al. [26]. Their study also linked HRR to smoking, diabetes, hypertension, hematocrit, uric acid, glucose, blood urea, creatinine, statin use, and younger age, none of which were evaluated in our study. These factors may explain differences in HRR proportions across studies, while the present findings suggest that HRR may have short-term prognostic relevance that warrants confirmation in larger, adjusted studies. The lack of detailed adjustment for diabetes, hypertension, renal function, smoking status, lipid profile, clinical presentation as acute coronary syndrome versus stable CAD, and other baseline cardiovascular risk factors further limits causal interpretation and prevents confirmation of HRR as an independent prognostic marker.

In our study, lower HRR levels were not significantly associated with symptomatic manifestations but were associated with mortality in the unadjusted analysis. Only four (2.9%) mortalities occurred, likely due to the shorter six-month follow-up compared with previous studies. Xiu et al. reported a nearly 5% mortality and a 13% MACE rate over approximately three years, with both MACE and mortality significantly associated with HRR [26]. Because mortality events were few in the present cohort, mortality-related estimates should be considered statistically unstable and hypothesis-generating. The low event count also precluded reliable multivariable mortality modeling.

Despite limitations in sample size and follow-up, the present findings suggest that HRR may have potential prognostic relevance in post-PCI patients; however, they do not establish HRR as an independent or externally validated predictor. In the present study, 21 (15.2%) patients had MHR levels above the selected cutoff, and elevated MHR was associated with mortality and mild symptoms in unadjusted analysis. This selected cutoff was derived from the present dataset and was not externally validated; therefore, the observed association should be interpreted with caution. Wu et al. [25] and Çiçek et al. [27] reported higher proportions of patients above the cutoff (75.2% and 74.8%, respectively) and found significant associations with age and other cardiovascular risk factors, which were not observed in our study.

Higher MHR may reflect greater inflammatory risk, suggesting that their populations may have included a larger proportion of clinically higher-risk patients. We also found MHR to be associated with short-term mortality in unadjusted analysis, which is directionally consistent with Çiçek et al. [27], who reported an association with in-hospital mortality. Both Wu et al. [25] and Çiçek et al. [27] observed that MHR was associated with long-term MACE and all-cause or cardiovascular mortality. In the present study, both MHR and HRR showed potential short-term prognostic relevance in exploratory unadjusted analyses. These results are directionally consistent with previous studies, but because the present cutoffs were internally derived and the study lacked an external validation cohort, their clinical generalizability remains uncertain. These findings are directionally consistent with observations from Wu et al. [25], Xiu et al. [26], and Çiçek et al. [27], although those studies included larger cohorts, longer follow-up, and more robust outcome assessment.

The present study has certain strengths. It used a prospective cohort design in a clinically relevant post-PCI CHD population and evaluated two simple, inexpensive, and routinely available laboratory-based indices. HRR and MHR can be calculated from commonly performed hematological and lipid parameters, which supports their potential practical relevance in resource-limited clinical settings. The study also attempted to compare the short-term prognostic behavior of two emerging markers and used ROC curve analysis as an exploratory method to assess their discriminatory performance. These strengths support the clinical relevance of the research question, but they should be interpreted in the context of the study’s methodological constraints.

A few methodological limitations should be considered while interpreting these findings. First, this was a single-center exploratory cohort study with a relatively small sample size and only four mortality events, limiting statistical power. Second, the six-month follow-up period restricted the assessment of long-term mortality, restenosis, repeat revascularization, stent thrombosis, recurrent myocardial infarction, and formal MACE. Third, the outcome category of mild symptoms was based on available clinical follow-up documentation and included symptoms such as fatigue, anxiety, or chest pain not requiring admission; therefore, some degree of subjectivity in outcome classification cannot be excluded. Fourth, blinded outcome adjudication was not performed. Fifth, detailed clinical presentation, procedural characteristics, renal function, diabetes status, medication adherence, and other established cardiovascular risk factors were not adjusted for in multivariable models. As a result, the associations observed for HRR and MHR should be interpreted as unadjusted short-term associations rather than evidence of independent prognostic value. Finally, the ROC-derived cutoffs were internally generated and require external validation before clinical use. These limitations reduce statistical robustness, external validity, and causal interpretability; therefore, the conclusions have been framed cautiously as preliminary and hypothesis-generating.

Future studies should include larger multicenter cohorts, longer follow-up periods, standardized endpoint definitions, formal adjudication of MACE, detailed procedural and pharmacological data, and adequately powered multivariable regression or survival analyses. Such studies are required to determine whether HRR and MHR provide independent and clinically meaningful prognostic information beyond established demographic, clinical, biochemical, and procedural predictors.

## Conclusions

The findings of this study suggest that MHR and HRR may have potential relevance as exploratory, routinely available laboratory indices associated with short-term post-PCI outcomes in CHD patients. In the present study, both markers showed short-term unadjusted associations with post-PCI outcomes, indicating their possible clinical relevance as easily available exploratory markers. However, it is important to acknowledge the major limitations of the study. First, the sample size was limited, and only four mortality events were recorded, which may have restricted the statistical power and generalizability of the findings. Second, the follow-up duration was short, preventing assessment of long-term outcomes and MACE. Additionally, the absence of a detailed cardiovascular risk profile for the patients was another limitation, as it restricted the ability to evaluate the predictive value of MHR and HRR in a multivariate context. Accordingly, the present findings should not be interpreted as definitive evidence that MHR and HRR are independent predictors of post-PCI outcomes. Similarly, the ROC-derived cutoff values should be considered internally derived and hypothesis-generating rather than clinically validated thresholds. Further studies involving larger sample sizes, longer follow-up periods, and comprehensive assessment of patient risk profiles are recommended to validate and substantiate the findings of the present study and to provide more robust, concrete, and reliable evidence regarding the prognostic relevance of these novel markers. Future multicenter studies should also include standardized endpoint definitions, formal adjudication of MACE, detailed procedural and pharmacological data, and adequately powered multivariable analyses to determine whether MHR and HRR provide independent prognostic information beyond established cardiovascular risk factors.
